# Crazed by Tobacco

**Published:** 1875-09

**Authors:** 


### CRAZED BY TOBACCO.

The Pulaski (Tenn). *Citizen* says: "We are extremely sorry to announce that the friends of Mr. Claude J. Woodring have been forced to take steps to deprive him of his liberty for a time, on account of the prevalent opinion among our people that his mind is over-balanced. His abberation is announced by physicians to be due to the excessive use of tobacco, and it was determined to confine him so that he could be effectually weaned from it. An inquisition was held at the County Court room Tuesday morning, and a verdict as above was reached."

This is only one case, and this one would never have seen the light of day but for the fact that the poisoned person chanced to be a man of prominence in his locality. Thousands of persons of lesser note have been destroyed by the insidious poison, and the world knows nothing of the fact. A day or two ago we were told of the case of a worthy citizen who evinced symptoms of mental derangement and presently became utterly insane. His delirium was wholly attributable to tobacco-smoking, the quantity consumed being about four cigars daily. This seems a small dose to many persons, but it is a very large dose for any one. If a person unused to the practice, were to consume such an amount of tobacco, it would probably kill him in three hours. Long continued use makes it possible to use this poison largely, just as the long continued use of opium or arsenic, permits an increased dose without fatal results. Still, these substances are poisons, virulent, dangerous poisons; and their poisonous effects exhibit themselves some time and in some way. Of one case of actual reported insanity, there are a hundred thousand cases of impaired intellects through the use of this terrible plant. Next to alcohol, tobacco, of all the substances known to man, can bear off the banner for destroying power. It has no single redeeming quality. Its influence is bad in every way. To say that it is *always* mentally, morally and physically lowering in its tendency and effects, is to state very mildly the facts. To say that the creature who consumes tobacco in any form, becomes thereby offensive to the rest of the world, is to utter the veriest truism. How any honorable man can indulge in such a dirty, destructive, demoralizing practice, merely because brain-sensibility is thereby somewhat deadened, has ever been a puzzle to us.

Tobacco is a poison and a disgusting poison. It may not kill for a time, but it will kill ultimately. During the killing process it makes of the subject operated on, a loathsome creature, to all persons of refined sensibility. Alcohol is not more deadly; strychnine, arsenic and opium, are not more carefully to be avoided, than this rank and powerful plant. It secures offspring of feeble bodies and feeble brains, and makes of strong men, imbeciles.

---

UNBOLTED WHEAT.—If you desire the most wholesome bread, you will use the best wheat, have it clean and coarsely ground, sift it not at all, and then cook it as follows: Take cold water—say one pint or one quart, according to the quantity one desires to make, or the size of the family demands—and with one hand sift in the unbolted flour through the fingers, stirring with the other till you have rather soft dough; then knead it for about five minutes; roll to about three-fourths of an inch thick; cut out with a common biscuit cutter and prick with a fork (this is done to prevent blistering on top) and place in a very hot oven, to bake thirty to forty-five minutes, according to the heat of your stove. Care should be taken not to burn the biscuit. This plan will make very light and sweet and wholesome biscuit.

